# Newly isolated *Lactobacillus paracasei* strain modulates lung immunity and improves the capacity to cope with influenza virus infection

**DOI:** 10.1186/s40168-023-01687-8

**Published:** 2023-11-23

**Authors:** Seungil Kim, Sohyeon Lee, Tae-Young Kim, Su-Hyun Lee, Sang-Uk Seo, Mi-Na Kweon

**Affiliations:** 1 Mucosal Immunology Laboratory, Department of Convergence Medicine, University of Ulsan College of Medicine/Asan Medical Center, Seoul, Republic of Korea; 2 Digestive Diseases Research Center, University of Ulsan College of Medicine, Seoul, Republic of Korea; 3 Department of Microbiology, College of Medicine, The Catholic University of Korea, Seoul, Republic of Korea

**Keywords:** Gut microbiota, *Lactobacillus paracasei*, IFN-I, Influenza

## Abstract

**Background:**

The modulation of immune responses by probiotics is crucial for local and systemic immunity. Recent studies have suggested a correlation between gut microbiota and lung immunity, known as the gut–lung axis. However, the evidence and mechanisms underlying this axis remain elusive.

**Results:**

In this study, we screened various *Lactobacillus (L.)* strains for their ability to augment type I interferon (IFN-I) signaling using an IFN-α/β reporter cell line. We identified *L. paracasei* (MI29) from the feces of healthy volunteers, which showed enhanced IFN-I signaling in vitro. Oral administration of the MI29 strain to wild-type B6 mice for 2 weeks resulted in increased expression of IFN-stimulated genes and pro-inflammatory cytokines in the lungs. We found that MI29-treated mice had significantly increased numbers of CD11c^+^PDCA-1^+^ plasmacytoid dendritic cells and Ly6C^hi^ monocytes in the lungs compared with control groups. Pre-treatment with MI29 for 2 weeks resulted in less weight loss and lower viral loads in the lung after a sub-lethal dose of influenza virus infection. Interestingly, IFNAR1^−/−^ mice did not show enhanced viral resistance in response to oral MI29 administration. Furthermore, metabolic profiles of MI29-treated mice revealed changes in fatty acid metabolism, with MI29-derived fatty acids contributing to host defense in a Gpr40/120-dependent manner.

**Conclusions:**

These findings suggest that the newly isolated MI29 strain can activate host defense immunity and prevent infections caused by the influenza virus through the gut–lung axis.

Video Abstract

**Supplementary Information:**

The online version contains supplementary material available at 10.1186/s40168-023-01687-8.

## Background

Commensal microbes play a pivotal role in shaping the immune system and defending the host against various pathogens through both local and systemic immune responses [1, 2]. In recent years, emerging evidence has illuminated the intricate crosstalk between the gut microbiota and the respiratory system, collectively known as the gut-lung axis [3, 4]. This dynamic interplay between these distant anatomical sites highlights the potential involvement of gut microbiota-derived metabolites in modulating protective immune responses in the lungs [5, 6]. Indeed, an increasing body of research suggests that the gut-lung axis plays a critical role in safeguarding against respiratory virus infections.

The human gut microbiota is a diverse community of microorganisms that coexist symbiotically with the host, contributing to various aspects of health and disease [7]. Among the myriad of gut microbial inhabitants, *Lactobacillus* spp. have attracted considerable attention for its beneficial effects on host health [8]. These lactic acid bacteria, including *Lactobacillus* and *Bifidobacterium*, are widely recognized as probiotics, used to promote gastrointestinal health and support the immune system [9]. Probiotics have been shown to inhibit the growth and activity of pathogenic microorganisms, including viruses, bacteria, and fungi, through mechanisms such as production of the metabolites and antimicrobial substances [10, 11]. While probiotics have been extensively studied for their role in the host’s defense against viral infections, particularly influenza virus, the underlying mechanisms remain incompletely understood.

In recent years, an increasing body of evidence has revealed a significant connection between the gut microbiota and lung immunity, suggesting potential shared mechanisms governing host defense in these seemingly distinct systems. Gut dysbiosis, resulting from factors such as antibiotic treatment, has been associated with worsened outcomes during influenza infection, indicating the crucial role of the gut microbiota in providing protection against respiratory viruses [12, 13]. Mechanistically, gut microbiota-derived metabolites, particularly short-chain fatty acids (SCFAs), have emerged as potential mediators of the gut-lung axis, influencing the lung’s ability to mount protective immune responses [14]. Additionally, specific gut-associated metabolites produced by certain bacterial species have been found to confer protection against respiratory virus infections, further reinforcing the concept of bidirectional communication between the gut and lungs.

Lipids, including fatty acids, represent an essential component of gut microbiota metabolism and exert profound effects on host physiology. Long-chain fatty acids, such as palmitic acid, have been identified as activators of Gpr40 and Gpr120, receptors involved in modulating glucose homeostasis and regulating various aspects of host metabolism [15]. Moreover, these fatty acids and their metabolites have been found to impact immune responses. For instance, they can enhance IgA production and influence the proliferation of specific immune cells in the gut [16, 17]. Interestingly, metabolites associated with linoleic acid, produced by *Lactobacillus* species, have been shown to modulate host metabolic conditions and inflammation by binding to Gpr40 and Gpr120 [18]. Given the multifaceted roles of gut microbiota-derived lipids, it is plausible that they may contribute to the regulation of immune responses in the lungs and influence the host’s ability to cope with respiratory infections.

Type I interferon (IFN-I) are antiviral substances produced by the host, primarily generated by plasmacytoid dendritic cells (pDCs), which act as specialized sensors of viral nucleic acids [19]. Once produced, IFN-I engages the IFN-α receptor (IFNAR) and triggers the transcription of interferon-stimulated genes (ISGs), encoding various proteins with antiviral functions [20, 21]. Studies have demonstrated the crucial role of the gut microbiota in controlling the constitutive production of IFN-I by pDCs and priming systemic antiviral immunity [22–24]. Additionally, IFN-I signaling plays a vital role in protecting against viral infections by promoting the generation of Ly6C^hi^ monocytes [25]. Inflammatory signals lead to the recruitment of Ly6C^hi^ monocytes at the site of infection, where they play an essential role in viral clearance [26]. Overall, emerging evidences suggest that the symbiotic gut microbiota modulate antiviral immunity through activation of IFN-I signaling.

In summary, our study explored the impact of gut microbiota on lung immunity and focused on a particular strain of *L. paracasei*. We aimed to investigate whether this strain could boost the host’s ability to defend against influenza infection by stimulating IFN-I signaling. The results demonstrated that the administration of *L. paracasei* enhanced the expression of ISGs and altered the fatty acid profile. Additionally, we discovered that *L. paracasei* protects the host from influenza infection through Gpr40 and Gpr120. These findings suggest that *L. paracasei* has promising potential to enhance the host’s defense immunity.

## Methods

### Ethics statement

All animal experiments were approved by the Institutional Animal Care and Use Committee of Asan Medical Center (Approval No. 2020–12-170). Fecal samples were collected from the human dock center of Asan Medical Center under the Institutional Review Board (Approval No. A20201614). All experiments were conducted under anesthesia using a combination of ketamine (100 mg/kg) and xylazine (20 mg/kg).

### Mice and virus infection

C57BL/6 (B6) mice were obtained from OrientBio (Seong-Nam, South Korea), and IFNAR^−/−^ mice (B6 background) were obtained from B&K Universal Ltd. (Hull, UK). The mice were 8–10-week-old females fed sterile food and water ad libitum housed in the animal facility of Asan Medical Center (Seoul, South Korea) and kept under specific pathogen-free conditions. They were anesthetized and infected intranasally with a sub-lethal dose (1 × 10^3^ PFU) of influenza A/PR8 in PBS. All experiments were conducted in accordance with the relevant ethical guidelines and regulations.

### Isolation of *Lactobacillus* and *Bifidobacterium* from human stool

A total of 86 fecal samples, taken from fresh residual samples after fecal occult blood and parasitic examination on the same date, were obtained from the human dock center of Asan Medical Center. Fecal samples were suspended in PBS and then seeded onto MRS agar (BD Bioscience) and TOS-MUP agar (Kisan-Bio). The fecal cultures were maintained at 37℃ under anaerobic conditions generated using a GasPak 100 system (BD Bioscience). Approximately 500 colonies were selected from MRS and TOS-MUP plates and tested using PCR for the *Lactobacillus* genus with the primer set 5′-AGCAGTAGGGAATCTTCCA-3′ and 5′-ATTYCACCGCTACACATG-3′, or the *Bifidobacterium* genus with the primer set 5′-TGGCAGTCCAACAAGCRC-3′ and 5′-TAGGAGCTCCAGATGCCGTG-3′.

### IFN-α/β reporter cell line culture

To screen for bacteria that enhance IFN-I signaling, we cultured an IFN-α/β reporter HEK cell line (Invivogen) in DMEM (Gibco) with 10% FBS (Gibco), 1% penicillin–streptomycin (Gibco), recombinant IFN-β (R&D Systems), and 4% bacterial culture supernatants. After overnight culture, we conducted a colorimetric assay using QUANTI-Blue solution (Invivogen) according to the manufacturer’s instructions. Bacterial culture supernatants were centrifuged at 8000 rpm for 10 min and passed through a 0.45-μm syringe filter (Pall Corp.). To investigate the effectiveness of fatty acids in enhancing IFN-I signaling, we added bovine serum albumin-conjugated palmitic acid (4 mM, Sigma) to the culture media of the IFN-α/β reporter HEK cell line. To inhibit Gpr40/120, we used a Gpr40 antagonist (GW1100, 100 µM; Cayman Chemical) and a Gpr120 antagonist (AH7614, 100 µM; Cayman Chemical).

### Oral administration of *L. paracasei*

*L. paracasei* MI3 and MI29 (KCTC 14636BP) were cultured in MRS and maintained in an anaerobic incubator using the GasPak 100 system at 37℃. The cultures were centrifuged, and the resulting pellet was suspended in anaerobic PBS. The pellet was orally administered to mice daily for 2 weeks using a Zonde needle at a dose of 1 × 10^9^ CFU per administration. For the comparison study, the type strain of *L. paracasei* was obtained from the Korean Collection for Type Cultures (KCTC 3510). To inhibit Gpr40/120, C57BL/6 mice were treated with intraperitoneal injections of Gpr40 antagonist (GW1100, 2.5 mg/kg/day; Cayman Chemical) and Gpr120 antagonist (AH7614, 2.5 mg/kg/day, Cayman Chemical) while also being orally administered the MI29 strain for 2 weeks.

### Cell isolation and flow cytometry analysis

Mouse lung tissues were diced using scissors and suspended in 5 mL of digestion buffer comprising RPMI-1640, 10% FBS, 2-mM l-glutamine, 1-mM sodium pyruvate, 20-mM HEPES, 1% penicillin–streptomycin, collagenase D (2 mg/mL; Roche), and DNase I (15 μg/mL; Roche). The samples were incubated for 1 h at 37℃ and then centrifuged. The resulting pellet was suspended in 1 mL of RBC lysis buffer and incubated for 1 min at room temperature. The cells were washed with PBS before use. The collected cells were first incubated with anti-CD16/32 antibody (BD Bioscience), followed by staining with a Live/Dead cell staining kit, anti-PDCA-1(eBio927) from Invitrogen; anti-CD45 (30-F11), anti-CD11b (M1/70) from BD Bioscience; anti-CD11c (N418), anti-Ly6C (HK 1.4); and anti-Ly6G (1A8) from BioLegend.

### Viral plaque assay

The entire lung was removed and homogenized to prepare lung extracts in PBS. The tissue samples were centrifuged at 12,000 rpm for 5 min, and the resulting supernatant was collected. MDCK cells cultured in MEM (Cytiva), supplemented with 10% FBS and 1% penicillin–streptomycin, were seeded at 1 × 10^6^ cells 1 day before analysis. The collected samples were incubated with PBS-washed cells for 1 h at room temperature. Thereafter, the plate was overlaid with MEM containing 1% low-melting agarose (Lonza) and 10 μg/mL of trypsin (Gibco) and incubated at 37℃, 5% CO_2_ for 3 days. The cells were fixed in 4% formalin overnight at room temperature, and the plaques were counted.

### Real-time PCR

Total RNA was extracted from lung tissues using the RNeasy Mini Kit, while total RNA from *L. paracasei* was extracted using Trizol (Thermo Fisher Scientific). cDNA was synthesized using the ReverTra Ace qPCR RT Master Mix with gDNA Remover (Toyobo). The cDNA was used as a template for real-time (RT) PCR, which was performed using SYBR green chemistry (Thermo Fisher Scientific) on an RT PCR system (Applied Biosystems). The following RT PCR primers were used in this study: *Oas1*, 5′-CGCACTGGTACCAACTGTGT-3′ and 5′-CTCCCATACTCCCAGGCATA-3′; *Cxcl10*, 5′-TTTCTGCCTCATCCTGCTG-3′ and 5′-CTCATCATTCTTTTTCATCGTG-3′; *Ifit1*, 5′-CAGAAGCACACATTGAAGAA-3′ and 5′-TGTAAGTAGCCAGAGGAAGG-3′; *Ifit3*, 5′-GCCGTTACAGGGAAATACTGG-3′ and 5′-CCTCAACATCGGGGCTCT-3′; *Rsad2*, 5′-AACAGGCTGGTTTGGAGAAG-3′ and 5′-TGCCATTGCTCACTATGCTC-3′; *Irf7*, 5′-GCCAGGAGCAAGACCGTGTT-3′ and 5′-TGCCCCACCACTGCCTGTA-3′; *Oasl2*, 5′-GGATGCCTGGGAGAGAATCG-3′ and 5′-TCGCCTGCTCTTCGAAACTG-3′; *Mx1*, 5′-AAGATGGTCCAAACTGCCTTCG-3′ and 5′-GCCTTGGTCTTCTCTTTCTCAGC-3′; *IL-1b*, 5′-GTTGACGGACCCCAAAAGAT-3′ and 5′-AAGGTCCACGGGAAAGACAC-3′; *IL-6*, 5′-ATGGATGCTACCAAACTGGA-3′ and 5′-TTGGATGGTCTTGGTCCTTA-3′; *IL-18*, 5′-GGCTGCCATGTCAGAAGACT-3′ and 5′-ATCTTCCTTTTGGCAAGCAA-3′; *accA*, 5′-GCACGCGGGAACTGATTAAC-3′ and 5′-TCGATGAAACTTGGCTGCCT-3′; *accB*, 5′-ATCTGGTCAAAGCAAGTGCG-3′ and 5′-AGTACGGATCAGCGTCAGGT-3′; *accC*, 5′-CTCTGTGGCGGTGTTCTCAA-3′ and 5′-AATCCGGCATCGGTTACGAG-3′; *accD*, 5′-AGCAAGGCAGCTATCGTGAG-3′ and 5′-CACCAGATCGGCATCCCATT-3′; *fabD*, 5′-CAGGTTGAAGCGTGGTTGTC-3′ and 5′-TTAGTCGGTTCAGCCACCTG-3′; *fabH*, 5′-CGAATCACAGTCCGTTTGCC-3′ and 5′-CTGGTGTTGCCATGCTCATC-3′; *fabZ*, 5′-CTTGGCGGCATTAAGAAGGC-3′ and 5′-TTGCCCAAACCTGCATTGTC-3′.

### RNA-seq analysis

A library of isolated total RNA was prepared using the TruSeq mRNA Sample Prep Kit (Illumina). PolyA-selected RNA extraction, RNA fragmentation, random hexamer-primed reverse transcription, and 100-nt paired-end sequencing were performed using the HiSeq4000 platform (Illumina). RNA data processing and statistical analysis were performed by Macrogen Inc. (Seoul, Korea). The pre-processed raw reads were aligned to the reference genome (mm10) using HISAT2 version 2.1.0. StringTie version 2.1.3b was used to assemble the aligned reads. Differentially expressed genes were defined as those with a *p* value of < 0.05 and a fold change of > 2. Gene enrichment and pathway analysis were performed based on Gene Ontology.

### Histology and cytokine detection

Lung tissues were dissected and fixed in 4% paraformaldehyde and subsequently embedded in paraffin. The tissue sections were then stained with hematoxylin and eosin (H&E). Pathology scoring was conducted in a blinded fashion using a scoring system based on the level of lung tissue destruction, epithelial cell layer damage, and polymorphonuclear cell infiltration. The levels of IFN-α and IFN-β in the lung tissues were measured by ELISA (R&D systems) according to the manufacturer’s protocol.

### Metabolome profiling

Metabolites were extracted from bacteria media, mouse lung, and cecum using conventional liquid–liquid extraction procedures. Briefly, 100 μL of bacteria media was mixed with 3–4 volumes of chloroform/methanol (1/2, v/v) and centrifuged for 15 min. For lung and cecum, 400 uL of chloroform/methanol (2/1, v/v) were added to tissues and homogenized using a TissueLyzer (Qiagen), followed by centrifugation for 15 min. An additional 100 uL of chloroform or water was added if phase separation could not be achieved. Nonpolar metabolites containing lipids were collected from the lower organic phase and polar metabolites from the upper aqueous phase. The organic or aqueous solution was dried using a vacuum centrifuge, then stored at − 20℃ until analysis. The dried matter of the organic or aqueous solution was reconstituted with 100 μL of 0.1% formic acid in methanol or 50% of LC mobile phase A, respectively, before LC–MS analysis. All solvents were purchased from Sigma-Aldrich or Thermo Fischer Scientific. LC–MS/MS was equipped with an Ultimate3000 (Dionex) and LTQ-OrbitrapXL (Thermo Fisher Scientific). A reverse-phase column (Pursuit 5; 150 × 2.0 mm) and HILIC column (Waters XBridge BEH amide; 150 × 2.1 mm) were used for nonpolar and polar metabolites, respectively. LC–MS/MS analysis was conducted for each sample solution in positive and negative ion modes. For a reverse-phase column, mobile phase A was 0.1% formic acid in H_2_O, and mobile phase B was 0.1% formic acid in methanol/isopropanol (85/15). The flow rate was 300 µL/min, and the column oven was set at 40℃. The separation gradient was as follows: 75 to 99.9% of B for 2 min, hold at 99.9% of B for 13 min, 99.9 to 75% of B for 0.5 min, and then hold at 75% of B for 4.5 min. For a HILIC column, mobile phase A was a 10-mM ammonium acetate, 10-mM ammonium hydroxide in 95% H_2_O/5% acetonitrile, and pH 9, and mobile phase B was a 10-mM ammonium acetate, 10-mM ammonium hydroxide in 5% H_2_O/95% acetonitrile, and pH 7. The flow rate was 300 µL/min, and the column oven was set at 40℃. The separation gradient was as follows: 70% of B for 1 min, 70 to 40% of B for 5 min, hold at 40% of B for 6 min, 40 to 70% of B for 0.1 min, and then hold at 70% of B for 7.9 min. Data normalization was performed using log transformation and auto-scaling. Metabolite features, including neutral mass and retention time values, were determined using Compound Discoverer 3.2. The metabolite features that displayed statistically significant changes (fold change > 1.2 or < 0.8, with *p* < 0.05) were selected and identified using the METLIN and KEGG database with a mass accuracy of 10 ppm. Statistical analyses, including ANOVA and heatmap generation, were conducted using MetaboAnalyst 5.0.

### Whole-genome sequencing

The gDNA of MI29 and MI3 strains was extracted using the MagAttract HMW DNA kit (Qiagen), following the manufacturer’s instructions. The integrity of the gDNA was assessed by running agarose gel electrophoresis, and the quantity was measured using the Qubit 2.0 fluorometer (Invitrogen). For shearing, 5 μg of genomic DNA was processed using the Megaruptor3, according to the manufacturer’s protocol. The SMRTbell library was prepared using the SMRTbell Express Template Preparation Kit (Pacbio). The genome and library size and Qubit concentration were entered into the calculator provided by Pacbio, and the library was pooled according to the numerical value. The SMRTbell library was sequenced using the Sequel Sequencing Kit v3.0 and SMRT Cell 1 M v2. The de novo genome assembly was constructed using the Pacbio sequencing data, and sequencing analysis was performed by CJ Bioscience, Inc. The gene-finding and functional annotation pipeline of the whole-genome assembly were carried out using the EzbioCloud genome database. Comparative whole-genome analysis was conducted using average nucleotide identity base BLAST (ANIb). The ANIb value was calculated with the ANI calculator from Kostas lab (http://enve-omics.ce.gatech.edu/ani). Operons, including *acc* and *fab*, were predicted by the Operon Mapper [27]. The phiSITE database confirmed the − 10 and − 35 regions in promoter sequences of these genes [28].

### Statistical analysis

Statistical analyses were performed using Prism software (GraphPad, La Jolla) with a two-tailed *t* test and one-way analysis of variance (ANOVA) followed by Tukey’s post hoc test. Data are presented as mean ± SD. *P* values of < 0.05, < 0.01, and < 0.001 were considered statistically significant.

## Results

### *L. paracasei* isolated from human feces induced IFN-I activity

**Fig. 1:**
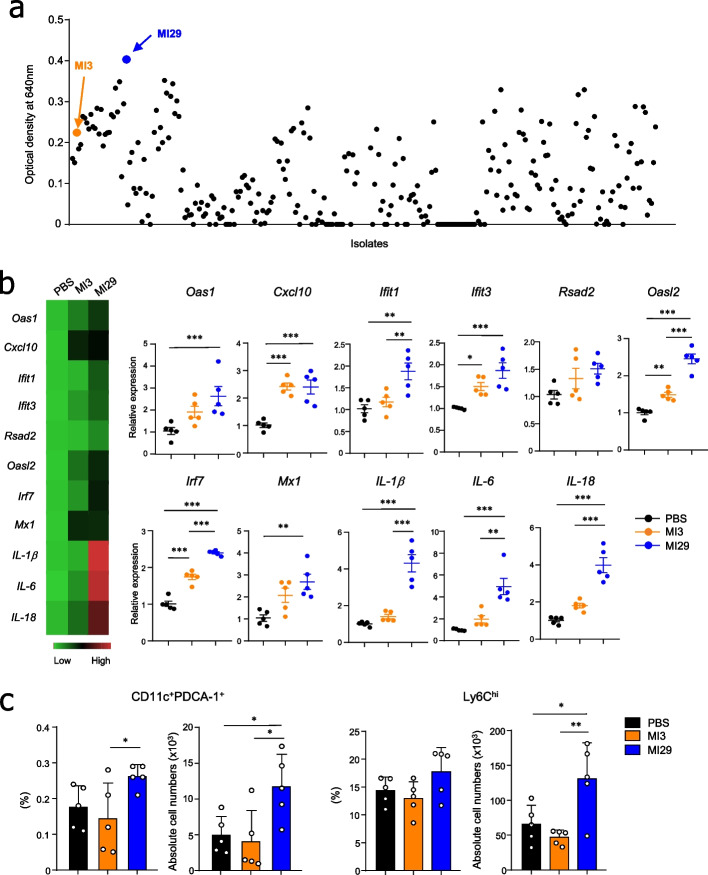
Screening of IFN-I-inducing bacterial strain from human feces. **a** Scatter plot displaying a colorimetric assay using an IFN α/β reporter cell line. **b** Heat map and mRNA levels for ISGs (*Oas1*, *Cxcl10*, *Ifit1*, *Ifit3*, *Rsad2*, *Oasl2*, *Irf7*, and *Mx1*) and pro-inflammatory cytokines (*IL-1β*, *IL-6*, and *IL-18*) from lung tissues. **c** Frequencies and absolute cell numbers of CD45^+^CD11c^+^PDCA-1^+^ and CD45^+^CD11b^+^Ly6G^−^Ly6C^hi^ cells in lung tissues. Statistical analyses were performed using one-way ANOVA with post hoc Tukey’s test. ^*^p < 0.05, ^**^p < 0.01, and ^***^p < 0.001. Data were combined from at least three independent experiments

By utilizing selective media, we were able to isolate strains of *Lactobacillus* and *Bifidobacterium* from fecal samples collected from healthy human volunteers. Our objective was to identify new bacterial strains that enhance IFN-I signaling. We screened over 200 strains using an IFN-α/β reporter cell line and observed the highest increase in reporter activity in the *L. paracasei* strain MI29 (Fig. 1a). MI3, an *L. paracasei* strain that exhibited low amplification of IFN-I signaling, was selected as the control (Fig. 1a).

### Oral administration of *L. paracasei* MI29 led to an increase in antiviral genes in the lungs

To determine whether IFN-I-inducing bacteria promote the transcription of ISGs, MI29 was orally administrated to mice for 2 weeks. MI29-treated mice showed an increased expression of ISGs (i.e., *Oas1*, *Cxcl10*, *Ifit1*, *Ifit3*, *Rsad2*, *Irf7*, *Oasl2*, and *Mx1*) in lung tissues compared with those treated with PBS or MI3 (Fig. 1b). Furthermore, pro-inflammatory cytokines (i.e., *IL-1β*, *IL-6*, and *IL-18*) were upregulated in the lungs of MI29-treated mice. Since IFN-I and IL-1β are mainly produced by pDCs and monocytes, respectively, during inflammation [29, 30], we next examined the populations of pDCs and monocytes in the lungs of PBS- or MI29-treated mice. MI29-treated mice had increased frequencies and numbers of pDCs and Ly6C^hi^ monocytes (Fig. 1c and Supplementary Fig. 1). Overall, these findings demonstrate that MI29 boosts host defense-related immune responses at steady state.

### Oral administration with *L. paracasei* MI29 strain protected the host against influenza infection

**Fig. 2:**
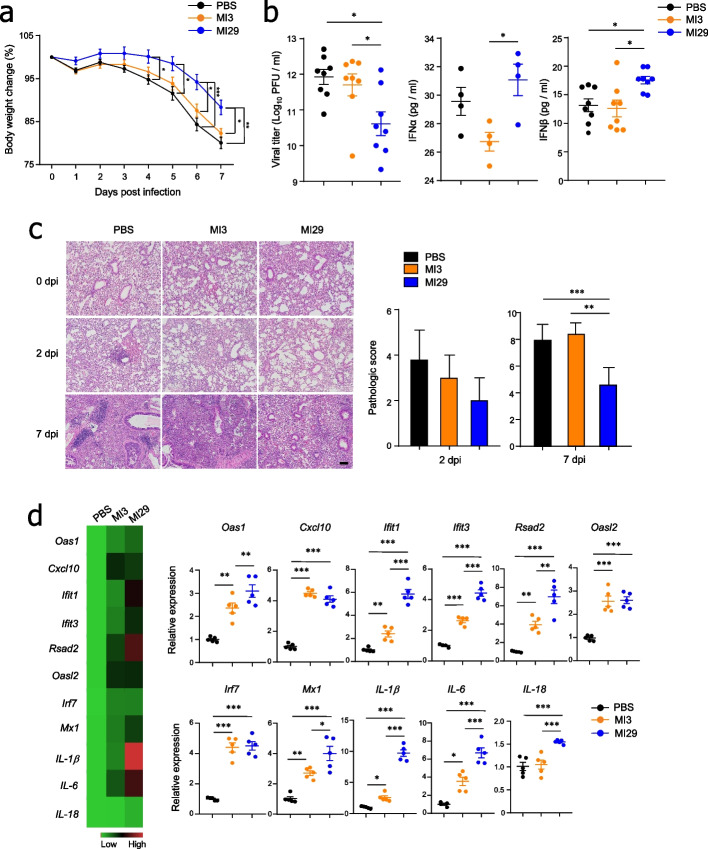
Oral administration of *L. paracasei* MI29 protects the host from influenza infection. **a** Changes in body weight of mice after A/PR8 infection. **b** Viral titers of lung tissue measured by plaque assay using the MDCK cell line and expression levels of IFN-I in lung homogenate measured by ELISA at 2 dpi. **c** Lung tissue pathology and scores determined by H&E staining. **d** Heat map and mRNA levels for ISGs (*Oas1*, *Cxcl10*, *Ifit1*, *Ifit3*, *Rsad2*, *Oasl2*, *Irf7*, and *Mx1*) and pro-inflammatory cytokines (*IL-1β*, *IL-6*, and *IL-18*) in lung tissues. Statistical analyses were performed using one-way ANOVA with post hoc Tukey’s test. ^*^p < 0.05, ^**^p < 0.01, and ^***^p < 0.001. Data are combined from at least three independent experiments

After demonstrating that MI29 boosts host defense-related immune responses at steady state, we investigated whether oral administration with MI29 protects against viral infection. We observed less weight loss and decreased viral titers in the lungs of MI29-treated mice compared with PBS- or MI3-treated mice after influenza virus infection (Figs. 2a, b). We also saw significant upregulation of IFN-α/β levels in the lung homogenates of mice treated with MI29 (Fig. 2b). Furthermore, compared with mice treated with PBS or MI3, those treated with MI29 orally exhibited less severe lung damage, including lung tissue destruction, epithelial cell layer damage, and polymorphonuclear cell infiltration (Fig. 2c). Gene ontology pathway enrichment analysis of lung tissues from MI29-treated mice revealed upregulation of genes related to inflammatory, defense response, and leukocyte migration pathways (Supplementary Fig. 2a). Adipokines such as Adipoq (adiponectin), Lep (leptin), and Orm1 (Orosomucoid1) were upregulated in the lung of MI29-treated mice compared with PBS- or MI3-treated mice (Supplementary Fig. 3a). Furthermore, MI29-treated mice exhibited higher levels of Fabp4 (fatty acid binding protein 4), Hamp (hepcidin antimicrobial peptide), and Trim55 (tripartite motif-containing protein 55) compared with mice treated with MI3 (Supplementary Fig. 3a). In the infectious state, mRNA levels of ISGs and pro-inflammatory cytokine genes increased in the lung of MI29-treated mice compared with PBS- or MI3-treated mice (Fig. 2d). Notably, transcripts associated with various chemokines (e.g., Ccl20, Cxcl2, Ccl11, and Ccl3) that fall under the gene ontology term “cellular response to IL-1” were highly expressed after MI29 treatment (Supplementary Figs. 2b and 3b). These results suggest that MI29 plays an essential role in activating protective immunity, which protects the host from future infection.

### *L. paracasei* MI29 strain promotes antiviral effects in an IFN-I-dependent manner

**Fig. 3:**
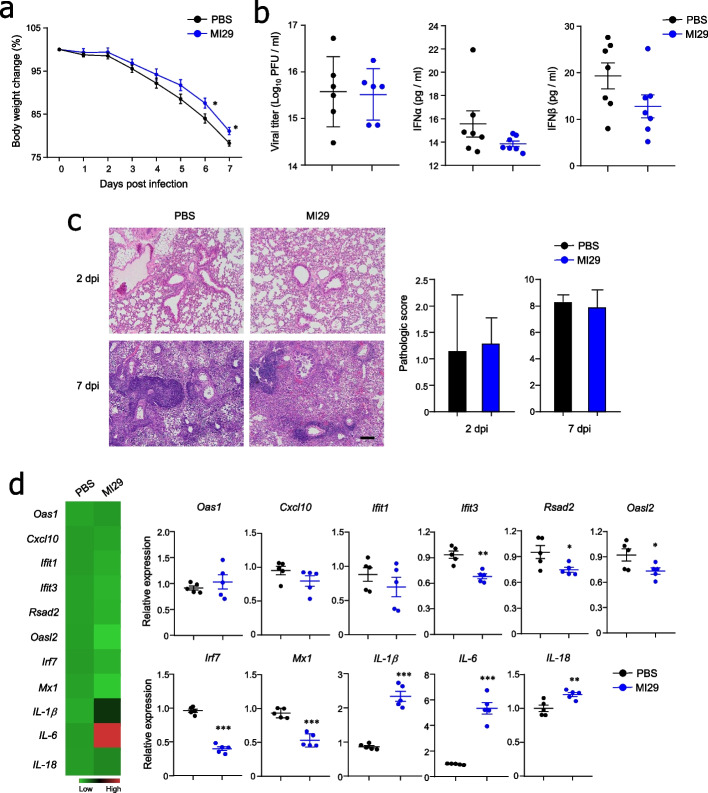
Administration of *L. paracasei* MI29 enhances IFN-I-dependent resistance to virus infection. **a** Body weight changes of mice after A/PR8 infection. **b** Plaque assay using MDCK cell line for viral titer of lung and expression level of IFN-I in lung homogenate using ELISA at 2 dpi. **c** Pathology and scores of lung tissues from H&E staining. **d** Heat map and mRNA levels for ISGs (*Oas1*, *Cxcl10*, *Ifit1*, *Ifit3*, *Rsad2*, *Oasl2*, *Irf7*, and *Mx1*) and pro-inflammatory cytokines (*IL-1β*, *IL-6*, and *IL-18*) from lungs at 2 dpi. Statistical analyses were performed using a two-tailed paired *t* test. ^*^p < 0.05, ^**^p < 0.01, and ^***^p < 0.001. Data are combined from at least three independent experiments

To evaluate the exact role of IFN-I in the antiviral effects of the MI29 strain, IFNAR1^−/−^ mice were utilized. Following influenza virus infection, MI29-treated mice had less body weight loss than PBS-treated IFNAR1^−/−^ mice (Fig. 3a). However, viral titers and levels of IFN-α/β were comparable in the lung homogenates of MI29- and PBS-treated IFNAR1^−/−^ mice (Fig. 3b). Histopathology analysis of lung tissues indicated that the protective effect of the MI29 strain was diminished in IFNAR1^−/−^ mice (Fig. 3c). Moreover, the expression levels of ISGs were not upregulated in MI29-treated IFNAR1^−/−^ mice compared with those treated with PBS (Fig. 3d). In contrast, pro-inflammatory cytokine genes increased in MI29-treated IFNAR1^−/−^ mice compared with PBS-treated mice (Fig. 3d). These results suggest that the protective role of the MI29 strain against influenza virus infection is primarily mediated by IFN-I.

### Oral administration of *L. paracasei* MI29 altered metabolic profiles

**Fig. 4:**
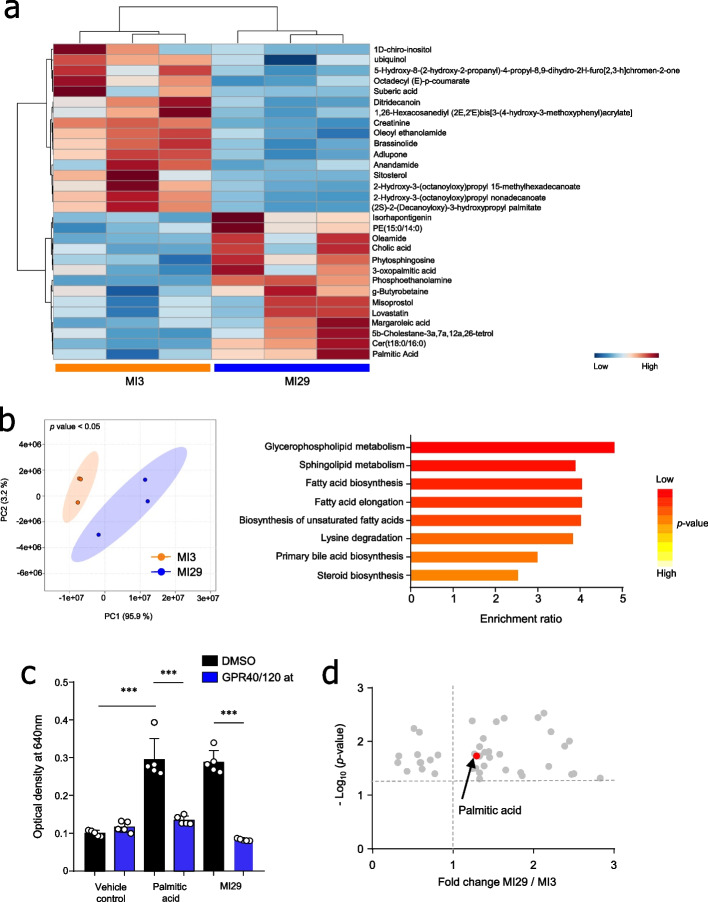
Metabolic profiles were altered by administration of *L. paracasei* MI29. **a** Heat map displaying the metabolic profiles in the cecum of mice treated with MI3 or MI29. **b** Principal component analysis plot showing clustering between *L. paracasei* MI3- and MI29-treated mice. The metabolite enrichment analysis summary is based on the metabolites that showed significant variation in the cecum contents from MI29-treated mice compared with MI3-treated mice. **c** Colorimetric assay using IFN-α/β reporter cell line with 4 mM of palmitic acid or culture supernatant of *L. paracasei* MI29 by the treatment of Gpr40/120 antagonist. **d** Non-targeted metabolite analysis of bacterial culture supernatant. Statistical analyses were performed using one-way ANOVA with post hoc Tukey’s test. ^*^p < 0.05, ^**^p < 0.01, and ^***^p < 0.001. Data are combined from at least three independent experiments

Considering the crucial role of microbiota-derived metabolites in the gut–lung axis, we conducted a non-targeted analysis to examine metabolites in both cecum contents and lung tissue. Among the annotated metabolites, 25 and 30 significantly differed in cecum contents and lung tissue between MI3- and MI29-treated mice (Fig. 4a and Supplementary Fig. 4a). PCA analysis revealed that the two groups of mice clustered separately (Fig. 4b and Supplementary Fig. 4b). To investigate functionally related metabolites, metabolite enrichment analysis was conducted. Notably, the fatty acid-related pathway was enriched in the cecum contents and lung tissue of MI29-treated mice compared with MI3-treated mice (Fig. 4b and Supplementary Fig. 4b). To confirm whether fatty acids affected IFN-I signaling, we treated an IFN-α/β reporter cell line with palmitic acid. Interestingly, both palmitic acid or the culture supernatant from the MI29 strain amplified IFN-I signaling; however, this effect was no longer observed when a Gpr40/120 antagonist was used (Fig. 4c). Through non-targeted metabolite analysis of bacterial culture supernatant, higher levels of palmitic acid were observed in the culture supernatant of MI29 than in MI3 (Fig. 4d). These results suggest that the administration of MI29 enriched fatty acid-related metabolites.

### *L. paracasei* MI29 strain predominantly expressed fatty acid synthesis

**Fig. 5:**
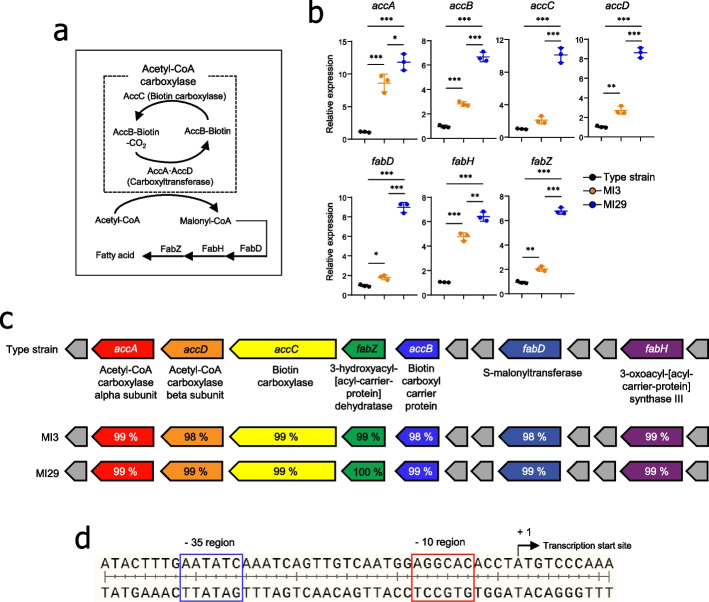
The expression of genes related to fatty acid synthesis increased in *L. paracasei* MI29. **a** Model of the metabolic pathway for the production of bacterial fatty acids. **b** mRNA expression levels of acetyl-CoA carboxylase (*acc*) and fatty acid biosynthesis (*fab*) compared between type, MI3, or MI29 strains. **c** Operon-containing gene for production of fatty acid. DNA sequences homogeneity (%) of *acc* and *fab* from MI3 and MI29 strains compared to type strain (*L. paracasei* KCTC 3510). **d** Promoter sequences of the operon for fatty acid synthesis. Statistical analysis was performed using one-way ANOVA with post hoc Tukey’s test. ^*^p < 0.05, ^**^p < 0.01, and ^***^p < 0.001. Data are combined from at least three independent experiments

Previous studies have demonstrated that fatty acid synthesis is essential for cell growth, as fatty acids are components of storage lipids and membrane phospholipids [31]. To further investigate how the MI29 strain produced more palmitic acid than the MI3 strain, we confirmed the expression levels of fatty acid synthesis-related genes, such as acetyl-CoA carboxylase (Acc) and fatty acid biosynthesis (Fab) [32, 33] (Fig. 5a). The mRNA expression levels of Acc (accA, accB, accC, and accD) and Fab (fabD, fabH, and fabZ) genes in the MI29 strain were higher than those in the type (KCTC 3510) and MI3 strains (Fig. 5b). We then assessed the operon containing the *acc-* and *fab*-coding genes to investigate whether the observed differences in mRNA expression were due to promoter sequence regulation of transcription. Notably, type strain and MI3 and MI29 strains had identical promoter sequences of the operon (Figs. 5c, d). To further characterize the novel MI29 strain, we conducted whole-genome sequencing to analyze its genetic characteristics, such as genome size, number of coding sequences, and ANIb (Supplementary Fig. 5a). Additionally, we compared the carbohydrate utilization and enzyme profiles of the two strains to analyze their metabolic patterns (Supplementary Fig. 5b and c). The MI29 strain showed similar carbohydrate utilization but fermented more d-maltose and amygdalin (Supplementary Fig. 5b). Regarding enzyme profile, the MI29 strain expressed higher levels of esterase lipase and Naphthol-AS-BI-phosphohydrolase but lower levels of β-galactosidase than the MI3 strain (Supplementary Fig. 5c). These findings suggest that the MI29 strain had superior fatty acid biosynthesis capabilities and a distinct metabolic profile compared with the MI3 strain.

### *L. paracasei* MI29 protected the host against influenza virus in a Gpr40/120-dependent manner

**Fig. 6:**
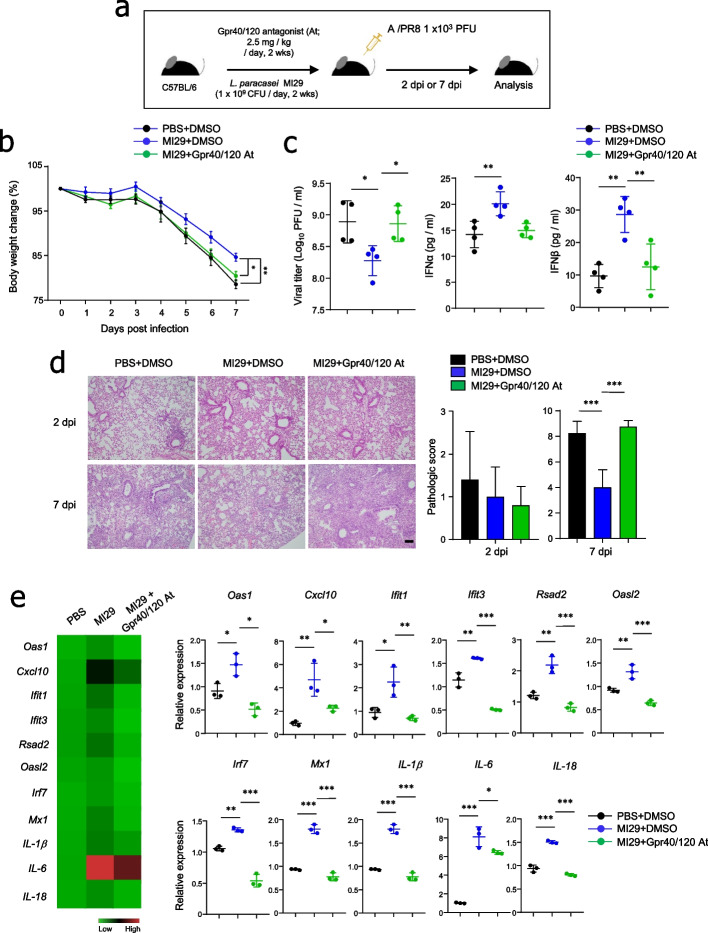
*L. paracasei* MI29 protects the host in a fatty acid-dependent manner. **a** Experimental scheme. **b** Body weight changes of mice after A/PR8 infection. **c** Plaque assay using MDCK cell line for viral titer of lung and expression levels of IFN-I in lung homogenate using ELISA at 2 dpi. **d** Pathology and scores of lung tissues by H&E staining. **e** Heat map and mRNA levels for ISGs (*Oas1*, *Cxcl10*, *Ifit1*, *Ifit3*, *Rsad2*, *Oasl2*, *Irf7*, and *Mx1*) and pro-inflammatory cytokines (*IL-1β*, *IL-6*, and *IL-18*) from lung at 2 dpi. Statistical analyses were conducted using one-way ANOVA with post hoc Tukey’s test. ^*^p < 0.05, ^**^p < 0.01, and ^***^p < 0.001. Data are combined from at least three independent experiments

We utilized a Gpr40/120 antagonist to investigate the effects of fatty acids on host defense against the MI29 strain. We simultaneously administered MI29 and the Gpr40/120 antagonist to wild-type B6 mice and infected them with a sub-lethal dose of influenza virus (Fig. 6a). The mice treated with MI29 plus Gpr40/120 antagonist exhibited a significant reduction in body weight compared with those treated with MI29 plus DMSO after influenza infection (Fig. 6b). Gpr40/120 antagonist treatment eliminated the protective immune responses induced by the MI29 strain, such as low viral load and high IFNα/β secretion (Fig. 6c). Histopathological analysis further supported the significant role of Gpr40/120-mediated signals in the MI29-mediated protective immunity against influenza virus infection (Fig. 6d). As expected, mRNA levels of ISGs and pro-inflammatory cytokine genes were significantly downregulated in the lungs of mice treated with MI29 plus Gpr40/120 antagonist compared with MI29 plus DMSO (Fig. 6e). Our findings therefore suggest that the MI29 strain protects the host from influenza virus infection in a Gpr40/120-dependent manner.

## Discussion

**Fig. 7:**
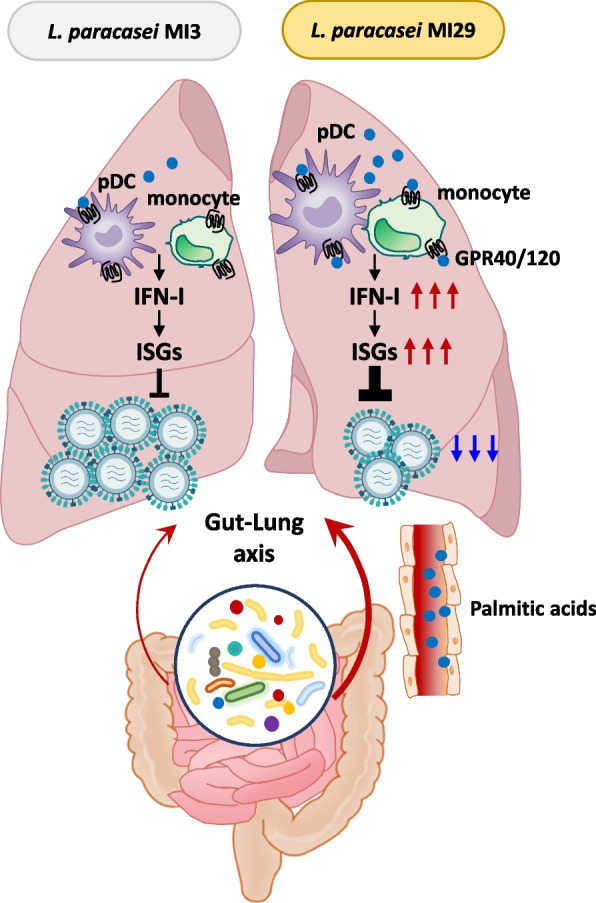
Scheme for the “gut–lung axis” by *L. paracasei* MI29 and its metabolites. *L. paracasei* plays a crucial role in immune activation and host defense against viral infection. *L. paracasei*-derived metabolites, such as palmitic acid, have a preventive effect against influenza virus infection

This study uncovers the crucial role of *L. paracasei* MI29 in protecting the host against viral infections by activating IFN-I signaling. The administration of *L. paracasei* MI29 led to an increase in both ISGs and fatty acid production, including palmitic acid. Remarkably, this elevation in fatty acids contributed to the host’s defense against influenza infection in a Gpr40/120-dependent manner. These findings shed light on a novel gut–lung axis, where *L. paracasei* MI29 stimulates fatty acids to enhance the immune response (see Fig. 7).

The gut microbiota provides immune-mediated protection via the gut–lung axis. For instance, germ-free mice infected with *Klebsiella pneumonia* were more susceptible to bacterial infection in an IL-10-dependent manner [34]. Moreover, SCFAs produced by bacteria fermentation of inulin increased population of patrolling monocyte and effector function of CD8^+^ T cell, thereby preventing influenza virus-associated lung pathology [5]. SCFAs act in the lung to protect against allergic inflammation by enhancing the hematopoiesis of dendritic cell precursors [14]. Additionally, bacterial-derived products, such as lipopolysaccharide, stimulate TLR signaling, restoring the migration of respiratory tract dendritic cells to draining lymph nodes and priming T cells in the lung of antibiotic-treated mice [13]. These findings, combined with our results, suggest that gut microbiota-derived metabolites can diffuse systemically and activate the immune response in the lung.

Our findings are consistent with reports in the literature on the regulation of IFN-I response by the microbiota, which confirmed that commensal microbiota induces IFN-I production and expression of ISGs [6, 22]. Several studies have reported that *Lactobacillus* induces IFN-I production and expression of ISGs [35]. In particular, lactic acid bacteria, such as *Lactococcus*, *Leuconostoc*, *Streptococcus*, and *Pediococcus*, have been shown to stimulate pDCs and IFN-I production in a TLR9- and Myd88-dependent manner [36]. Based on these studies and our findings, we speculate that *Lactobacillus* may modulate pDC populations and have a strain-dependent capacity for inducing IFN-I signaling.

Extensive studies have been conducted on probiotics for respiratory and digestive virus infections to elucidate clinical clues [37–39]. Bacteria-derived metabolites are a possible antiviral mechanism of the gut microbiota, among which fatty acids may play a crucial role in regulating metabolic conditions and the immune response. Evidence strongly suggests that palmitic acids can activate the immune system through several ways. For instance, they stimulate pro-inflammatory responses by releasing IL-1β, IL-6, and TNF-α in the serum and also lead to increased inducible nitric oxide synthase levels in macrophages [40, 41]. Palmitic acids also act as a TLR ligand of human monocyte-derived dendritic cells [42]. Additionally, treatment of human T cells with palmitic acids induces expression of the lymphocyte-activating molecule and inflammatory cytokines [43]. Regarding IFN-I, palmitic acids induce IFN-I responses in murine hepatocytes and macrophages [44]. In this study, given that mice were fed the same normal chow diet, we hypothesized that palmitic acids produced by MI29 contributed to the enhancement of IFN-I signaling and resistance to influenza virus infection in vivo. Taken together, we conclude that palmitic acid plays an important role in immune activation and prevents influenza virus-induced symptoms of infection.

Previous studies have reported that various immune cells such as pDCs, monocytes, macrophages, and T cells, along with lung epithelial cells, express Gpr40/120, which is associated with the regulation of immune responses [45, 46]. For instance, *Clostridium butyricum*-induced ω-3 fatty acids and 18-eicosapentaenoic acid enhance IFN-λ production through Gpr120 signaling in lung epithelial cells, ameliorating influenza virus infection [47]. Additionally, ω-3 treatment decreases intraperitoneal macrophage chemotaxis by acting through Gpr120 [48]. In the liver, free fatty acids activate Gpr120, reducing the Th1 response and increasing the Treg population [46]. Furthermore, α-linolenic acid-derived metabolites from lactic acid bacteria modulate macrophage differentiation by acting through Gpr40 [49]. Combining these findings with our results, it is evident that lipid-like metabolites, acting through Gpr40/120 signaling, play a critical role in modulating innate and acquired immune responses.

IL-1β is a critical cytokine for the host’s defense response against infections and is produced by innate immune cells in response to pathogen-associated and damage-associated molecular patterns [50]. Inflammasome activation regulates IL-1β production, and saturated fatty acids such as palmitic acid promote NLRP3, which leads to the secretion of IL-1β [51, 52]. Moreover, IL-1β production is stimulated by pathogens and probiotics such as *Lactobacillus* and *Bifidobacterium* [53]. In this study, *L. paracasei* MI29 was found to increase IL-1β expression in the lung tissues of IFNAR^−/−^ mice; however, it had only a minor protective effect of host infected with influenza virus (Fig. 3d). Further research is needed to understand the complex interactions of IL-1β in this context.

We initially expected the promoter sequence to exhibit slight variations between the MI29 and MI3 strains, considering the former expressed more genes related to fatty acid synthesis. However, our results revealed that the promoter sequence was remarkably conserved. While the promoter is indeed a crucial factor for transcription initiation, bacterial transcription initiation is regulated by a wide range of mechanisms including transcription factors, sigma factors, DNA methylation, and small regulatory RNA, all of which influence on the efficiency and frequency of this process [54]. The primary role of these mechanisms is to ensure that gene expression is appropriately controlled, allowing cells to respond to changes in the environment and cellular requirements. Consequently, it is plausible to speculate that even with identical promoter sequences, the expression levels of a gene may vary between two different strains.

## Conclusions

Our study indicates that the newly isolated *L. paracasei* MI29 strain can have a beneficial impact in host defense against viral infections. To our knowledge, this is the first study to demonstrate the effect of *L. paracasei* MI29-derived metabolites, specifically palmitic acids, on preventing influenza virus infection. Our study suggests a novel gut–lung axis through *L. paracasei* MI29 and fatty acids, which boost immune response. Our study can provide a valuable basis for identifying and evaluating human-derived probiotics for their potential health benefits.

### Supplementary Information

**Additional file 1: Fig. S1.** Administration of *L. paracasei* MI29 promotes the population of pDCs and monocytes. Flow cytometry analysis was performed to identify pDCs gated from CD45^+^CD11c^+^PDCA-1^+^cells and monocytes gated from CD45^+^CD11b^+^Ly6G^−^ cells in lung tissues. **Fig. S2.** Oral administration of *L. paracasei* MI29 promotes host defense-related pathways in the lung from WT or A/PR8-infected mice. (a) Gene ontology assignments of differentially expressed genes significantly altered in the lungs from *L. paracasei* MI29-treated mice versus PBS or *L. paracasei* MI3-treated mice. (b) Gene ontology assignments of differentially expressed genes significantly altered in the lungs of A/PR8-infected mice treated with *L. paracasei* MI29 compared to those treated with PBS or *L. paracasei* MI3. Upregulated genes are shown for specific pathways of interest. **Fig. S3.** Oral administration of *L. paracasei* MI29 promotes the expression of genes related to the defense pathway in the lungs of WT or A/PR8-infected mice. (a) Volcano plot shows the log_2_-fold change in gene expression in the lungs of *L. paracasei* MI29-treated mice versus PBS or *L. paracasei* MI3-treated mice. (b) Volcano plot shows the log_2_ fold-change in gene expression in the lungs of A/PR8-infected mice treated with *L. paracasei* MI29 compared with those treated with PBS or *L. paracasei* MI3. **Fig. S4.** Metabolic profiles in the lung tissues from *L. paracasei*-treated mice. (a) Heat map displays the metabolic profiles in the lung tissues of mice treated with MI3 or MI29. (b) The principal component analysis plot shows the clustering between the mice treated with *L. paracasei* MI3 and MI29. A summary of metabolite enrichment analysis is provided based on the metabolites that showed significant variation in the lung tissues of MI29-treated mice compared with MI3-treated mice. **Fig. S5.** Characterization of *L. paracasei* to determine a novel strain. (a) Summary of genome annotation. Profiling of carbohydrate fermentation (b) and enzyme activity (c).

## Data Availability

All sequencing data are publically available in the NCBI BioProject ID PRJNA953094 and PRJNA953153.

## Abbreviations

*L. paracasei*: *Lactobacillus paracasei*
SCFA: Short-chain fatty acid
Gpr: G-protein-coupled receptor
IFN: Interferon
pDC: Plasmacytoid dendritic cell
ISG: Interferon-stimulated gene
Ly6: Lymphocyte antigen-6
IL-1β: Interleukin-1 beta
