# Supplementary material for: Newly isolated Lactobacillus paracasei strain modulates lung immunity and improves the capacity to cope with influenza virus infection
Source: Microbiome. 2023 Nov 23;11:260. doi: 10.1186/s40168-023-01687-8 (PMC10666316; doi:10.1186/s40168-023-01687-8)
Supplement: Supplementary file 2 — Additional file 1: Fig. S1. Administration of L. paracasei MI29 promotes the population of pDCs and monocytes. Flow cytometry analysis was performed to identify pDCs gated from CD45+CD11c+PDCA-1+cells and monocytes gated from CD45+CD11b+Ly6G− cells in lung tissues. Fig. S2. Oral administration of L. paracasei MI29 promotes host defense-related pathways in the lung from WT or A/PR8-infected mice. (a) Gene ontology assignments of differentially expressed genes significantly altered in the lungs from L. paracasei MI29-treated mice versus PBS or L. paracasei MI3-treated mice. (b) Gene ontology assignments of differentially expressed genes significantly altered in the lungs of A/PR8-infected mice treated with L. paracasei MI29 compared to those treated with PBS or L. paracasei MI3. Upregulated genes are shown for specific pathways of interest. Fig. S3. Oral administration of L. paracasei MI29 promotes the expression of genes related to the defense pathway in the lungs of WT or A/PR8-infected mice. (a) Volcano plot shows the log2-fold change in gene expression in the lungs of L. paracasei MI29-treated mice versus PBS or L. paracasei MI3-treated mice. (b) Volcano plot shows the log2 fold-change in gene expression in the lungs of A/PR8-infected mice treated with L. paracasei MI29 compared with those treated with PBS or L. paracasei MI3. Fig. S4. Metabolic profiles in the lung tissues from L. paracasei-treated mice. (a) Heat map displays the metabolic profiles in the lung tissues of mice treated with MI3 or MI29. (b) The principal component analysis plot shows the clustering between the mice treated with L. paracasei MI3 and MI29. A summary of metabolite enrichment analysis is provided based on the metabolites that showed significant variation in the lung tissues of MI29-treated mice compared with MI3-treated mice. Fig. S5. Characterization of L. paracasei to determine a novel strain. (a) Summary of genome annotation. Profiling of carbohydrate fermentation (b) and enzyme act [file 40168_2023_1687_MOESM1_ESM.pptx]

## Slide 1
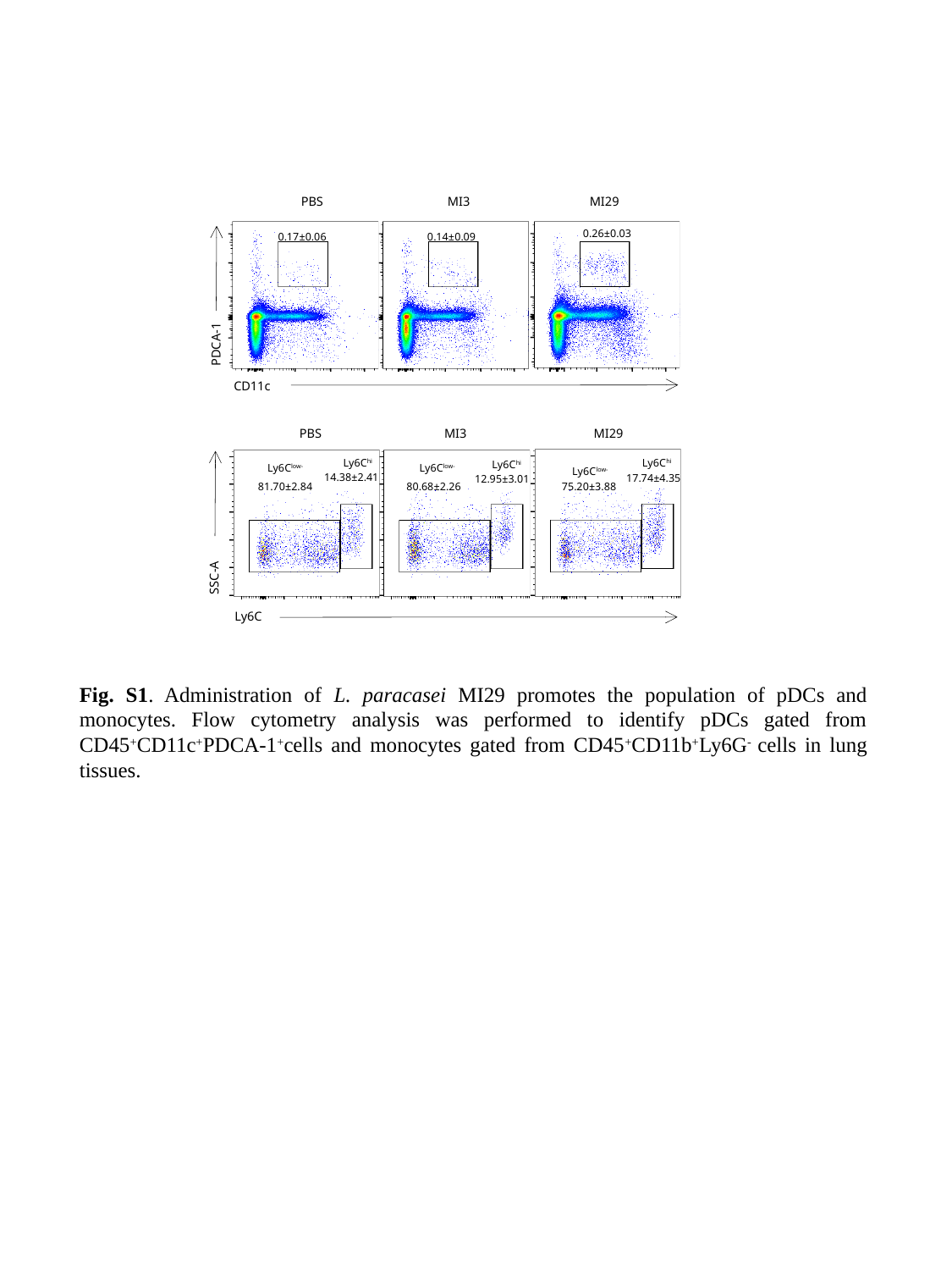

PBS
MI3
MI29
0.26±0.03
0.17±0.06
0.14±0.09
PDCA-1
CD11c
PBS
MI3
MI29
Ly6Chi
Ly6Chi
Ly6Chi
Ly6Clow-
Ly6Clow-
Ly6Clow-
14.38±2.41
17.74±4.35
12.95±3.01
75.20±3.88
81.70±2.84
80.68±2.26
SSC-A
Ly6C
Fig. S1. Administration of L. paracasei MI29 promotes the population of pDCs and monocytes. Flow cytometry analysis was performed to identify pDCs gated from CD45+CD11c+PDCA-1+cells and monocytes gated from CD45+CD11b+Ly6G- cells in lung tissues.

## Slide 2
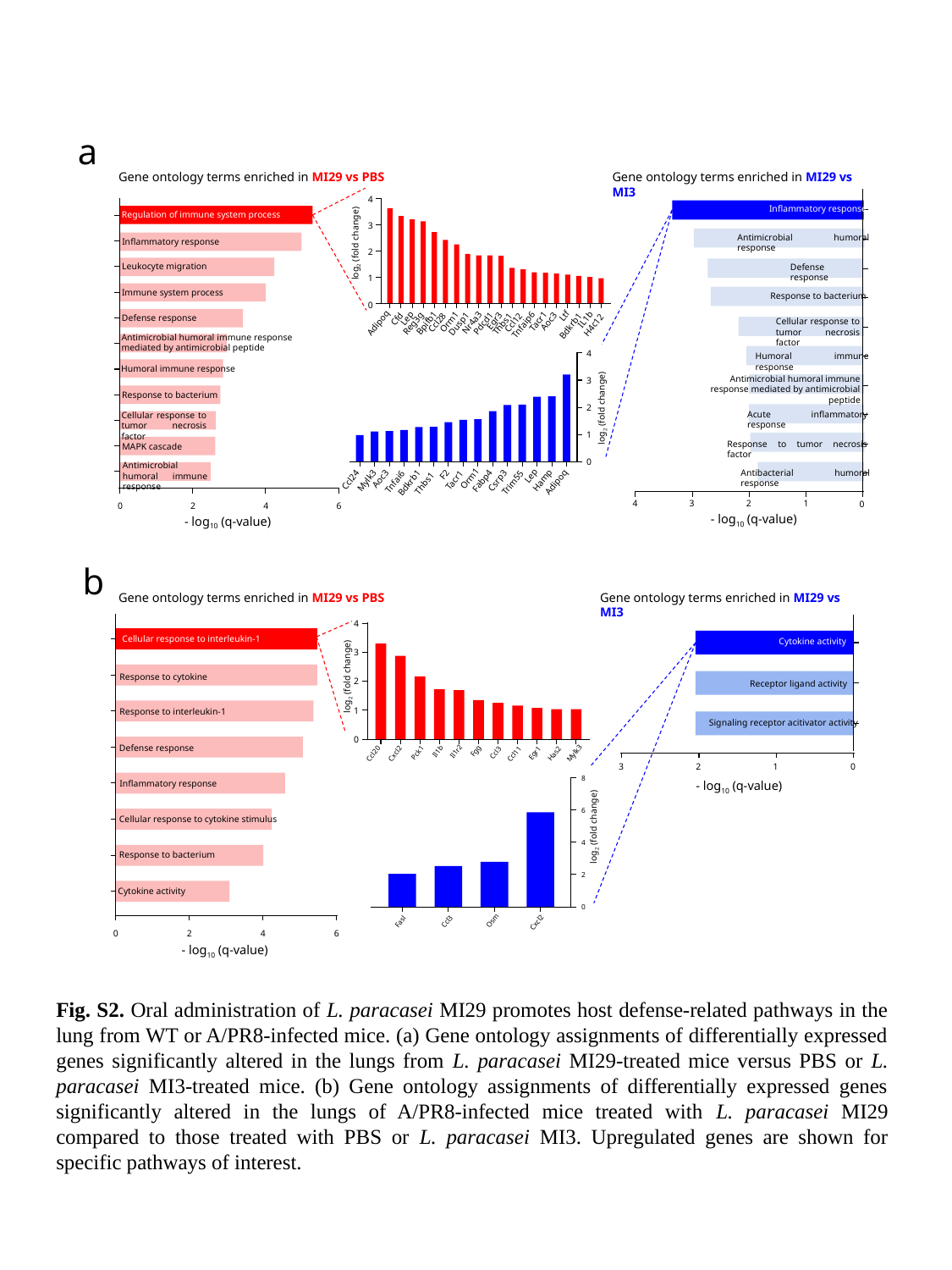

a
Gene ontology terms enriched in MI29 vs PBS
Gene ontology terms enriched in MI29 vs MI3
4
Inflammatory response
Regulation of immune system process
3
 log2 (fold change)
Antimicrobial humoral response
Inflammatory response
2
Leukocyte migration
Defense response
1
Immune system process
Response to bacterium
0
Ccl28
Ltf
Lep
IL1b
Cfd
Defense response
Egr3
Tacr1
Reg3g
Bpifb1
Nr4a3
Ccl12
Thbs1
Bdkrb1
Aoc3
Dusp1
Pdcd1
Tnfaip6
Adipoq
Orm1
H4c12
Cellular response to tumor necrosis factor
Antimicrobial humoral immune response mediated by antimicrobial peptide
4
Humoral immune response
Humoral immune response
Antimicrobial humoral immune response mediated by antimicrobial peptide
3
Response to bacterium
 log2 (fold change)
2
Acute inflammatory response
Cellular response to tumor necrosis factor
1
Response to tumor necrosis factor
MAPK cascade
0
Antimicrobial humoral immune response
F2
Lep
Antibacterial humoral response
Aoc3
Tacr1
Orm1
Hamp
Ccl24
Tnfai6
Mylk3
Csrp3
Fabp4
Adipoq
Bdkrb1
Thbs1
Trim55
4
3
2
1
0
0
2
4
6
- log10 (q-value)
- log10 (q-value)
b
Gene ontology terms enriched in MI29 vs PBS
Gene ontology terms enriched in MI29 vs MI3
4
Cellular response to interleukin-1
Cytokine activity
3
 log2 (fold change)
Response to cytokine
2
Receptor ligand activity
1
Response to interleukin-1
Signaling receptor acitivator activity
0
Defense response
Fgg
Il1b
Il1r2
Ccl3
Egr1
Pck1
Has2
Cxcl2
Ccl20
Ccl11
Mylk3
3
2
1
0
- log10 (q-value)
8
Inflammatory response
6
 log2 (fold change)
Cellular response to cytokine stimulus
4
Response to bacterium
2
Cytokine activity
0
Fasl
Osm
Ccl3
Cxcl2
0
2
4
6
- log10 (q-value)
Fig. S2. Oral administration of L. paracasei MI29 promotes host defense-related pathways in the lung from WT or A/PR8-infected mice. (a) Gene ontology assignments of differentially expressed genes significantly altered in the lungs from L. paracasei MI29-treated mice versus PBS or L. paracasei MI3-treated mice. (b) Gene ontology assignments of differentially expressed genes significantly altered in the lungs of A/PR8-infected mice treated with L. paracasei MI29 compared to those treated with PBS or L. paracasei MI3. Upregulated genes are shown for specific pathways of interest.

## Slide 3
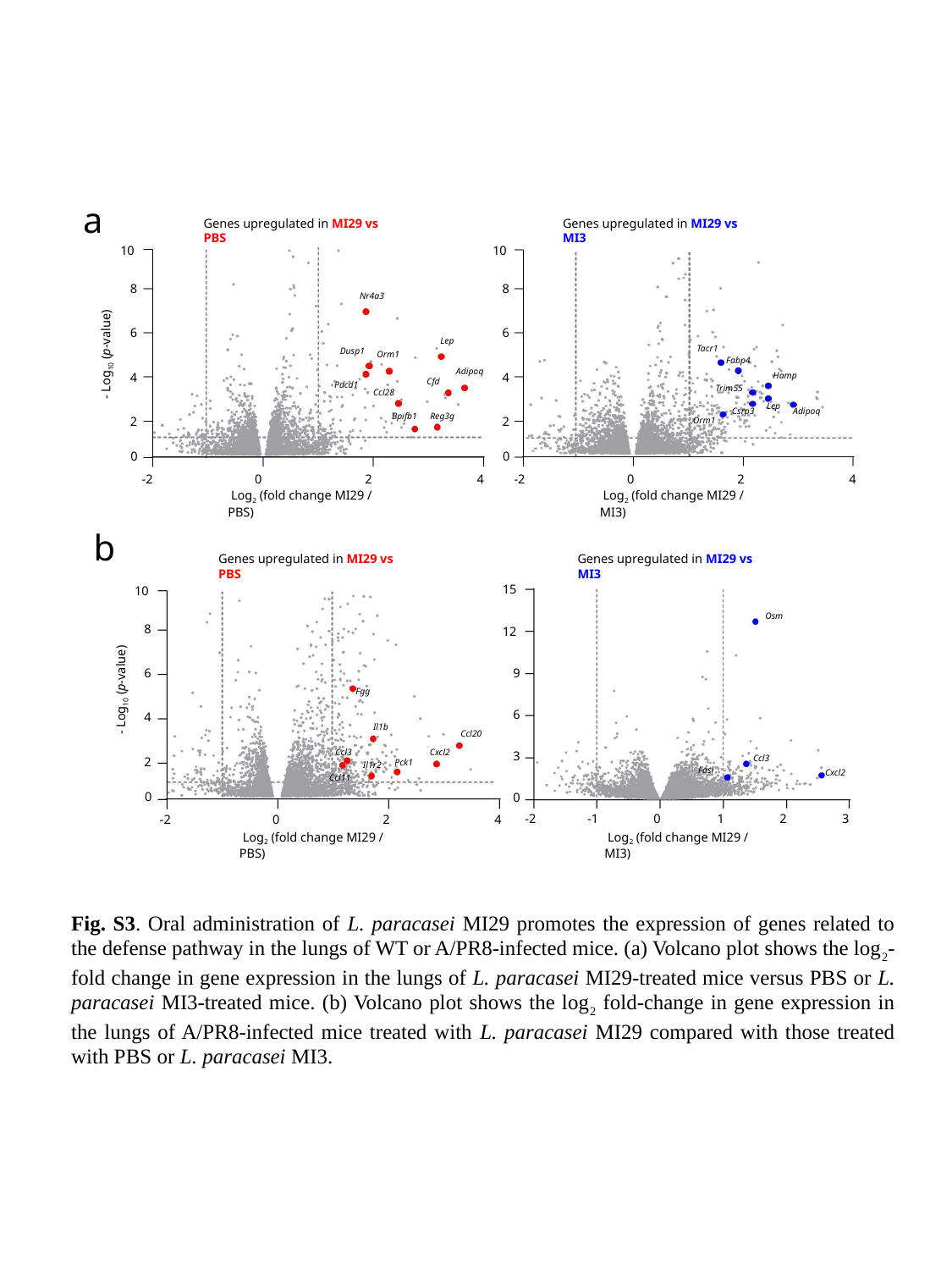

a
Genes upregulated in MI29 vs PBS
Genes upregulated in MI29 vs MI3
10
10
8
8
Nr4a3
6
6
Lep
- Log10 (p-value)
Tacr1
Dusp1
Orm1
Fabp4
Adipoq
Hamp
Cfd
4
4
Pdcd1
Trim55
Ccl28
Lep
Csrp3
Adipoq
Bpifb1
Reg3g
Orm1
2
2
0
0
-2
0
2
4
-2
0
2
4
 Log2 (fold change MI29 / PBS)
 Log2 (fold change MI29 / MI3)
b
Genes upregulated in MI29 vs PBS
Genes upregulated in MI29 vs MI3
15
10
Osm
8
12
9
6
- Log10 (p-value)
Fgg
6
4
Il1b
Ccl20
Ccl3
Cxcl2
Ccl3
3
Pck1
Il1r2
2
Fasl
Cxcl2
Ccl11
0
0
-2
-1
0
1
2
3
-2
0
2
4
 Log2 (fold change MI29 / PBS)
 Log2 (fold change MI29 / MI3)
Fig. S3. Oral administration of L. paracasei MI29 promotes the expression of genes related to the defense pathway in the lungs of WT or A/PR8-infected mice. (a) Volcano plot shows the log2-fold change in gene expression in the lungs of L. paracasei MI29-treated mice versus PBS or L. paracasei MI3-treated mice. (b) Volcano plot shows the log2 fold-change in gene expression in the lungs of A/PR8-infected mice treated with L. paracasei MI29 compared with those treated with PBS or L. paracasei MI3.

## Slide 4
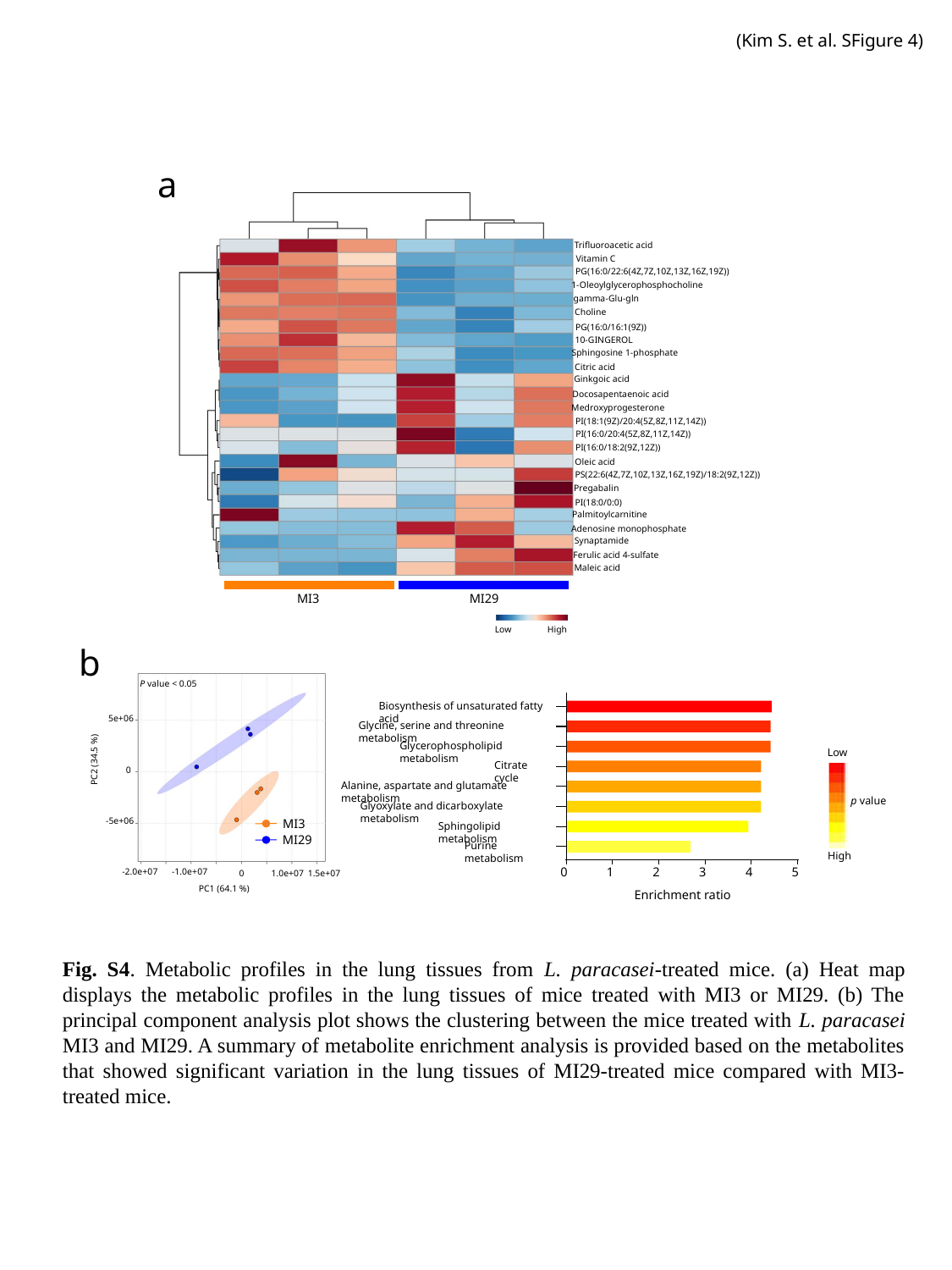

(Kim S. et al. SFigure 4)
a
Trifluoroacetic acid
Vitamin C
PG(16:0/22:6(4Z,7Z,10Z,13Z,16Z,19Z))
1-Oleoylglycerophosphocholine
gamma-Glu-gln
Choline
PG(16:0/16:1(9Z))
10-GINGEROL
Sphingosine 1-phosphate
Citric acid
Ginkgoic acid
Docosapentaenoic acid
Medroxyprogesterone
PI(18:1(9Z)/20:4(5Z,8Z,11Z,14Z))
PI(16:0/20:4(5Z,8Z,11Z,14Z))
PI(16:0/18:2(9Z,12Z))
Oleic acid
PS(22:6(4Z,7Z,10Z,13Z,16Z,19Z)/18:2(9Z,12Z))
Pregabalin
PI(18:0/0:0)
Palmitoylcarnitine
Adenosine monophosphate
Synaptamide
Ferulic acid 4-sulfate
Maleic acid
MI3
MI29
Low
High
b
P value < 0.05
0
1
2
3
4
5
Biosynthesis of unsaturated fatty acid
Glycine, serine and threonine metabolism
5e+06
Glycerophospholipid metabolism
Low
Citrate cycle
PC2 (34.5 %)
0
Alanine, aspartate and glutamate metabolism
Glyoxylate and dicarboxylate metabolism
p value
Sphingolipid metabolism
-5e+06
 MI3
 MI29
Purine metabolism
High
-2.0e+07
-1.0e+07
0
1.0e+07
1.5e+07
Enrichment ratio
PC1 (64.1 %)
Fig. S4. Metabolic profiles in the lung tissues from L. paracasei-treated mice. (a) Heat map displays the metabolic profiles in the lung tissues of mice treated with MI3 or MI29. (b) The principal component analysis plot shows the clustering between the mice treated with L. paracasei MI3 and MI29. A summary of metabolite enrichment analysis is provided based on the metabolites that showed significant variation in the lung tissues of MI29-treated mice compared with MI3-treated mice.

## Slide 5
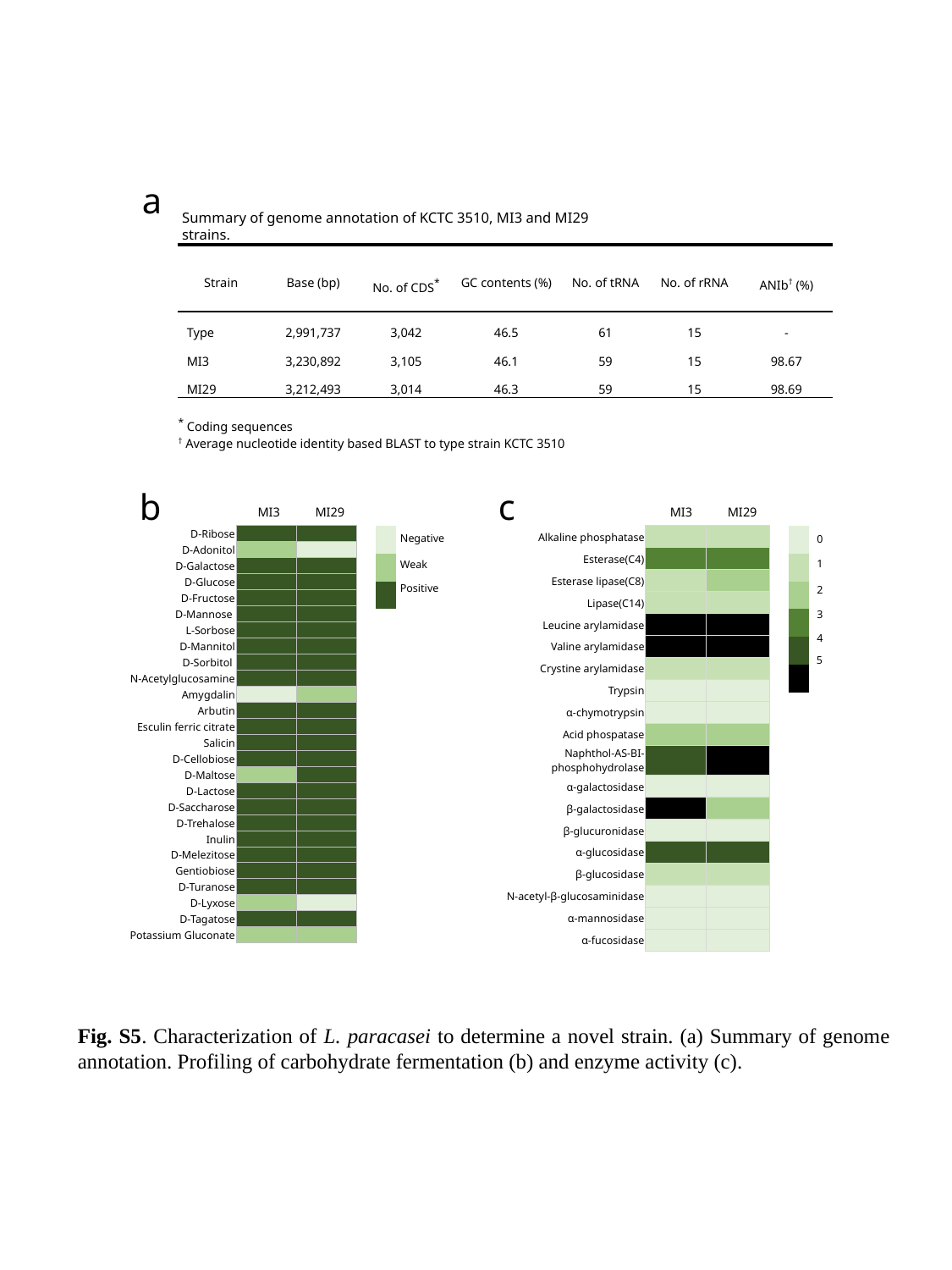

a
Summary of genome annotation of KCTC 3510, MI3 and MI29 strains.
| Strain | Base (bp) | No. of CDS\* | GC contents (%) | No. of tRNA | No. of rRNA | ANIb† (%) |
| --- | --- | --- | --- | --- | --- | --- |
| Type | 2,991,737 | 3,042 | 46.5 | 61 | 15 | - |
| MI3 | 3,230,892 | 3,105 | 46.1 | 59 | 15 | 98.67 |
| MI29 | 3,212,493 | 3,014 | 46.3 | 59 | 15 | 98.69 |
* Coding sequences
† Average nucleotide identity based BLAST to type strain KCTC 3510
b
c
MI3
MI29
MI3
MI29
Negative
| Alkaline phosphatase | | |
| --- | --- | --- |
| Esterase(C4) | | |
| Esterase lipase(C8) | | |
| Lipase(C14) | | |
| Leucine arylamidase | | |
| Valine arylamidase | | |
| Crystine arylamidase | | |
| Trypsin | | |
| α-chymotrypsin | | |
| Acid phospatase | | |
| Naphthol-AS-BI-phosphohydrolase | | |
| α-galactosidase | | |
| β-galactosidase | | |
| β-glucuronidase | | |
| α-glucosidase | | |
| β-glucosidase | | |
| N-acetyl-β-glucosaminidase | | |
| α-mannosidase | | |
| α-fucosidase | | |
| D-Ribose | | |
| --- | --- | --- |
| D-Adonitol | | |
| D-Galactose | | |
| D-Glucose | | |
| D-Fructose | | |
| D-Mannose | | |
| L-Sorbose | | |
| D-Mannitol | | |
| D-Sorbitol | | |
| N-Acetylglucosamine | | |
| Amygdalin | | |
| Arbutin | | |
| Esculin ferric citrate | | |
| Salicin | | |
| D-Cellobiose | | |
| D-Maltose | | |
| D-Lactose | | |
| D-Saccharose | | |
| D-Trehalose | | |
| Inulin | | |
| D-Melezitose | | |
| Gentiobiose | | |
| D-Turanose | | |
| D-Lyxose | | |
| D-Tagatose | | |
| Potassium Gluconate | | |
| |
| --- |
| |
| |
| |
| --- |
| |
| |
| |
| |
| |
0
1
Weak
Positive
2
3
4
5
Fig. S5. Characterization of L. paracasei to determine a novel strain. (a) Summary of genome annotation. Profiling of carbohydrate fermentation (b) and enzyme activity (c).
